# Individual and cohort-specific gut microbiota patterns associated with tissue-specific insulin sensitivity in overweight and obese males

**DOI:** 10.1038/s41598-020-64574-4

**Published:** 2020-05-05

**Authors:** Gerben D. A. Hermes, Dorien Reijnders, Ruud S. Kootte, Gijs H. Goossens, Hauke Smidt, Max Nieuwdorp, Ellen E. Blaak, Erwin G. Zoetendal

**Affiliations:** 1 Laboratory of Microbiology, Wageningen University & Research, 6708 WE Wageningen, The Netherlands; 2 TI Food and Nutrition (TIFN), P.O. Box 557, 6700 AN Wageningen, The Netherlands; 3 Department of Human Biology, NUTRIM School of Nutrition and Translational Research in Metabolism, Maastricht University Medical Center+, 6229 ER Maastricht, The Netherlands; 4 Department of Vascular Medicine and Department of Internal Medicine, University of Amsterdam, 1100 DD Amsterdam, The Netherlands

**Keywords:** Microbiome, Symbiosis, Prognostic markers, Metabolic syndrome, Pre-diabetes

## Abstract

A growing body of evidence suggests that the human gut microbiota plays a role in the development of obesity and related metabolic diseases. However, there is little consensus between studies, which could be due to biological as well as technical variation. In addition, little human data are available to investigate whether tissue-specific insulin sensitivity is related to specific microbial patterns. We examined this relation in two independent cohorts of overweight and obese pre-diabetic men, using phylogenetic microarray data and hepatic, peripheral and adipose tissue insulin sensitivity that were determined by a two-step hyperinsulinemic-euglycemic clamp with [6,6-^2^H_2_]-glucose tracer infusion. Despite a prominent subject-specific microbiota, we found significant associations of microbial taxa with tissue-specific insulin sensitivity using regression analysis. Using random forests we found moderate associations with other measures of glucose homeostasis in only one of the cohorts (fasting glucose concentrations AUC = 0.66 and HbA_1c_ AUC = 0.65). However, all findings were cohort-specific due to pronounced variation in microbiota between cohorts, suggesting the existence of alternative states for dysbiosis in metabolic syndrome patients. Our findings suggest individual or group related dynamics, instead of universal microbiota signals, related to the host when the overweight or obese state has already developed and argue that care should be taken with extrapolating significant correlations from single cohorts, into generalized biological relevance.

## Introduction

There is increasing evidence to suggest that our gut microbiome is associated with the development of obesity, insulin resistance and type 2 diabetes. This concept was first described in studies, in which germ-free mice showed less adiposity, improved insulin sensitivity and glucose tolerance as compared to conventionalized mice^1–4^. Later studies showed that the microbiota composition differs between lean, obese and diabetic mice and humans^5–8^. Microbial analyses of large genome-wide association studies showed that patients with type 2 diabetes are characterized by a decrease in the abundance of universal butyrate-producers and an increase in various opportunistic pathogens^5,6,9^. Nevertheless, between studies there is an overall lack of consistency regarding the identified microbial biomarkers and putative mechanisms underlying the observations. Discrepancies between studies could be a result of the heterogeneity of groups, genetic background, habitual lifestyle, diet and the methodology used by different researchers^10^. Meta-analyses have shown that individuals could be classified based on their microbiota as lean or obese with statistically significant accuracy within a study, consistent with the observation that this phenotype can be experimentally transferred in mice by microbiota transplantation^8^. However, microbial signatures were not consistent between studies even when the data were analysed in the same way^11^. Furthermore, the comparison of discriminant metagenomic markers for type 2 diabetes in European women^5^ and Chinese individuals^9^ revealed cohort specific differences, and the authors concluded that metagenomic predictive tools for type 2 diabetes should be specific for the age and geographical location of the populations studied^5^. Correspondingly, another putative marker for obesity based on the gut microbiome, the Bacteroidetes to Firmicutes ratio (B:F) proposed by Ley *et al*.^7^, remains controversial as it has been both refuted^12^ and contradicted^13^.

Nevertheless, transplantation of the microbiota in rodents and humans has provided evidence for the causal role of the microbiota in adiposity and metabolic health. Fecal transplantation from obese into germ-free mice significantly increased adiposity^8,14^, and fecal transplantation from lean donors into metabolic syndrome patients altered the recipients’ microbiota composition with a concomitant, minor improvement in peripheral insulin sensitivity based on responders and non-responders^15^. These observations were confirmed in a larger population, however the effect was also shown to be transient. Nevertheless, the authors identified a bacterial signature at baseline that was predictive for responder status^16^. In contrast, it has recently been demonstrated that interference with adult microbiota by 7-day antibiotic treatment has no clinically relevant impact on host metabolism in obese humans, despite deviant microbiota^17^. Importantly, the relationship between gut microbiota and tissue-specific insulin action has never been established in obese humans. Here, we investigated the relationship between the gut microbiota composition and adipose tissue, muscle and liver insulin sensitivity by means of the gold-standard two-step hyperinsulinemic-euglycemic clamp with [6,6-^2^H_2_]-glucose tracer infusion in two independent Dutch cohorts of overweight and obese pre-diabetic males from the Maastricht (MAA) and Amsterdam (AMS) region of the Netherlands.

## Results

### Subject characteristics

The subjects’ characteristics, including insulin resistance measurements are shown in Table 1. Subjects in both the MAA and AMS cohorts were insulin resistant (Homeostatic model assessment of insulin resistance (HOMA-IR): 4.5 ± 0.2 and 5.1 ± 0.3 respectively, ns). MAA presented with higher fasting glucose concentrations (6.1 ± 0.01 *vs*. 5.8 ± 0.09 mmol/l, p < 0.05), whereas insulin concentrations were lower than in AMS cohort (16.8 ± 0.8 *vs*. 20.0 ± 1.2 mU/l, p < 0.05). Homeostatic model assessment for beta-cell function (HOMA-B) was lower in MAA than in AMS (136.3 ± 7.5 *vs*. 192.0 ± 15.3%, p = 0.001). From the MAA cohort, 2 subjects had missing data for glycated haemoglobin (HbA_1c_), 2 for: insulin-mediated glucose disposal (Rd), 6 for % suppression of Endogenous Glucose Production (EGP) and 1 for % suppression of free fatty acids (FFA). For the AMS cohort there were no missing variables.

**Table 1:** Subjects’ characteristics.

	MAA (n = 56) Maastricht	AMS (n = 42) Amsterdam
Age	59.1 ± 1.0	54.9 ± 1.1
Weight (kg)	96.5 ± 1.3	116.3 ± 2.0
BMI (kg/m^2^)	31.2 ± 0.4	34.8 ± 0.5
Waist/hip ratio	0.95 ± 0.01	1.05 ± 0.01
Fasting insulin (mU/ml)	16.80 ± 0.79	20.02 ± 1.24
Fasting glucose (mmol/l)	6.06 ± 0.07	5.76 ± 0.09
HOMA-IR	4.5 ± 0.2	5.1 ± 0.3
HOMA-B%	136.3 ± 7.5	192.0 ± 15.3
HbA1c (%)	5.58 ± 0.05	5.74 ± 0.05
Fasting TAG	1.30 ± 0.10	1.50 ± 0.11
Rd (umol*kg^-1^*min^-1^)*	23.34 (10.7–51.4)	26.1 (10.1–40.0)
Suppression EGP (%)*	44.1(17.4–79.1)	55.6 (30.8–85.0)
Suppression FFA (%)*	45.3(−6,1–84.1)	74.4(53.9–92.1)

HOMA-IR: homeostasis model assessment for insulin resistance, HOMA-B%: homeostasis model assessment for beta-cell function, HbA1c: glycated haemoglobin, TAG: triacylglycerol, Rd: rate of disappearance, EGP: endogenous glucose production by the liver, FFA: free fatty acids. Data are expressed as mean ± SEM. Clamp-results are expressed as mean (range). *due to differences in clamp procedures (see Methods) between centers, no statistical comparison was made between cohorts.

Notably, clamp procedures were slightly different between centers (see Methods), and thus, potential differences in peripheral, hepatic and adipose tissue insulin sensitivity values between cohorts were not assessed.

### Fecal microbiota composition

**Figure 1:**
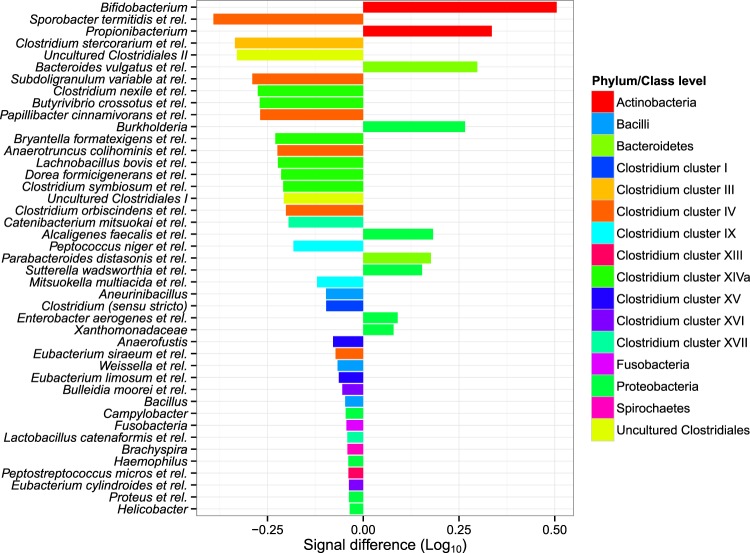
Enrichment of bacterial taxa in two separate cohorts of obese men. Genus like bacterial groups which showed significantly different abundance (Log_10_ signal intensity) between the two cohorts. The left side shows taxa enriched in AMS right side taxa enriched in MAA.

Remarkably, the average microbiota composition in both cohorts showed pronounced differences (Fig. 1). While Actinobacteria (*Bifidobacterium* and *Propionibacterium*) were more abundant in the MAA cohort, mainly genus-like groups of *Clostridium* clusters IV and XIVa were more abundant in the AMS cohort. More specifically, *Sporobacter termitidis* et rel. (IV), *Papillibacter cinnamivorans* et rel. (IV), *Subdoligranulum variable* et rel. (IV), *Anaerotruncus colihominis* et rel. (IV), *Butyrivibrio crossotus* et rel. (XIVa) and *Clostridium symbiosum* et rel. (XIVa), of which the latter four are known to contain butyrate-producing species, were more abundant in AMS than in MAA (Fig. 1). In addition, Uncultered *Clostriales* I and II were more abundant in AMS. A complete overview of the differential abundance of all detected taxa can be found in supplementary table 1.

**Figure 2:**
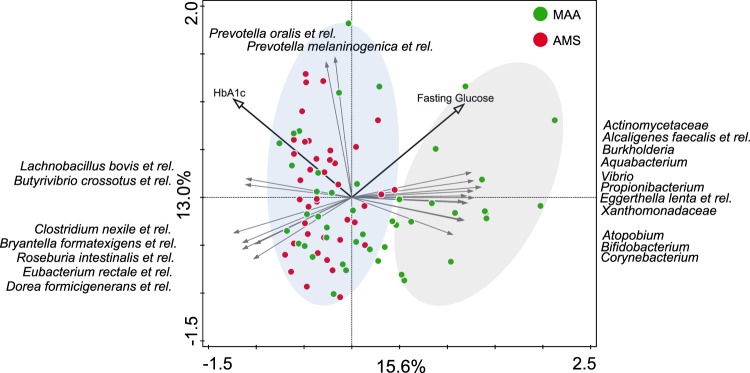
Principle component analysis of the fecal microbiota composition of 85 overweight insulin resistant overweight males from Maastricht (MAA) and Amsterdam (AMS). Individuals from AMS and a subset from MAA overlap and a second group of individuals in MAA was observed as indicated by the right ellipse. These also show associations with the two metabolic parameters associated with microbiota composition in MAA through Random Forests analysis. The direction of the species arrows depicts the abundance of microbial groups. Length of the arrows is a measure of fit. The environmental variable arrows approximate the correlation between species and an environmental variable. The further a sample falls in the direction indicated by the arrow, the higher the correlation. Samples near the coordinate origin (zero point) suggest near zero correlation.

Principal component analysis (PCA) analysis of the microbiota composition and calculation of within cohort Pearson correlations demonstrated that the variation between subjects from MAA was significantly higher than those from AMS (<2.2e-16, two-sided t-test, Supplementary Fig. 1) (Fig. 2). Although the majority of subjects from MAA overlapped in composition with those of AMS, it is remarkable that approximately 1/3 of MAA showed a distinct composition from this group along the first principal component. This explains the remarkable difference average microbiota composition between the two cohorts. Moreover, it suggests that some metabolic syndrome patients from MAA exhibit an alternative state of microbiota composition compared to the overlapping AMS and MAA individuals.

### Correlations between microbiota composition and host metabolic parameters

#### Tissue-specific insulin sensitivity

**Figure 3:**
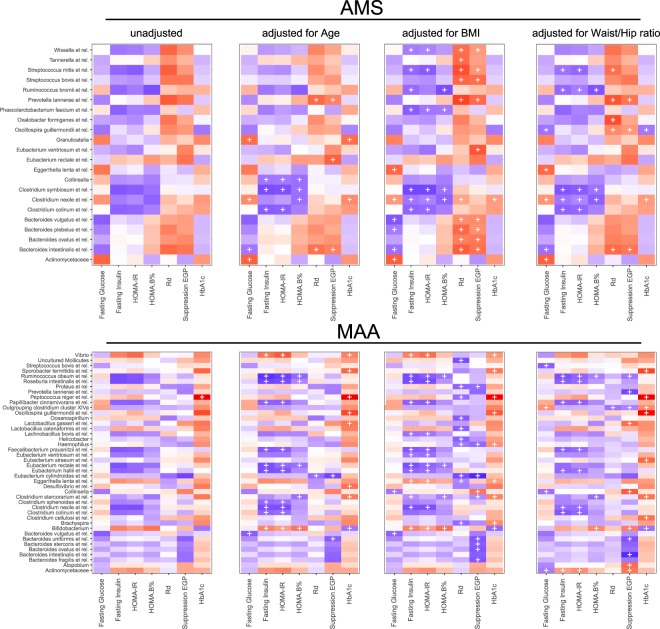
Correlation-heatmaps of host metabolic parameters and microbiota abundance. Heatmaps of (partial) Spearman correlations of tissue-specific insulin sensitivity and other markers of glucose homeostasis with individual genus like bacterial groups for AMS and MAA. Spearman correlations were adjusted for age, BMI and waist/hip ratio. Blue show negative correlations and red positive. A ‘ + ’ depicts correlations with a corrected p-value of q < 0.2.

After correction for multiple testing, peripheral, hepatic and adipose tissue insulin sensitivity (Rd, % suppression of EGP and % suppression of FFA, respectively), did not significantly correlate (q < 0.2) with the abundance of bacterial taxa at the genus like level in either cohort (Fig. 3). However, when adjusted for age, body mass index (BMI) and waist/hip ratio, several taxa did significantly correlate with Rd and % suppression of EGP in both cohorts (Fig. 3). Overall, the number of significant taxa with significant associations was higher in MAA. For both cohorts the number of significant correlations was dependent on the covariate, but in AMS all significant correlations were positive, while in MAA they were mostly negative, except for *Atopobium*, *Actinomycetaceae*, *Collinsella*, *Bifidobacterium* and *Lactobacillus gasseri* et rel. correlating with hepatic insulin sensitivity (% suppression of EGP). None of these associations were replicated in the other cohort, while several groups from the Bacteroidetes Phylum; *Prevotella tannerae* et rel*., Bacteroides spp* (*B. vulgatus*, *B. intestinales* and *B. ovatus*) showed an opposite association in both cohorts (positive in AMS, negative in MAA). Moreover, the overall pattern of correlations of specific taxa with tissue-specific insulin sensitivity was distinctly different in both cohorts (Fig. 3).

In addition, we determined non-linear multivariate relationships between the microbiota composition and tissue-specific insulin sensitivity. To this end, we ordered each dataset in quartiles of insulin sensitivity (Rd, %EGP and %FFA) and used random forest classifiers to determine whether the bacterial composition was related to these markers of tissue-specific insulin sensitivity, by using the highest and lowest quartiles as classes (supplementary Table 2). Both cohorts showed random classification for peripheral, liver and adipose tissue insulin sensitivity, indicating no significant relationship between microbial profiles and tissue-specific insulin sensitivity (data not shown).

#### Other measures of glucose homeostasis and insulin sensitivity

Except for the correlation of *Peptococcus niger* et rel. with HbA_1c_ (ρ 0.57, p = 5.52E-05, q = 0.06) in the MAA cohort, no significant correlation (q < 0.2) was found between abundance of specific microbial groups and HOMA-IR, HOMA-B, fasting glucose or insulin concentrations in either of the two cohorts (Fig. 3). However, again after correcting for age, BMI or waist/hip ratio several taxa did significantly correlate with other measures of glucose homeostasis. This time some significant correlations were replicated in both cohorts: *Clostridium colinum* et rel. negatively correlated with HOMA-IR and *B. vulgatus* et rel. negatively with fasting glucose. However, the same number of significant associations showed an opposite trend; *Actinomycetaceae* with fasting glucose (positive in AMS and negative in MAA) and *Oscillospira guillermondii* et rel. with HbA1c (negative in AMS and positive in MAA). Besides the replicated and opposite associations, the overall pattern of correlation of specific taxa with measures of glucose homeostasis was distinctly different in both cohorts (Fig. 3).

**Figure 4:**
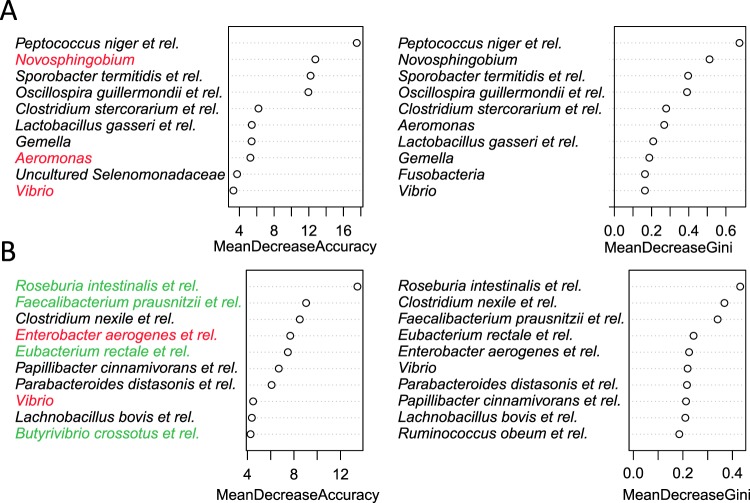
Top ten genus level groups with predictive power in classifying patients from MAA into the lowest and highest quartile of HbA_1c_ (A) and fasting glucose (B). The higher the group the more the prediction power will be reduced when the specific group is removed from the Random Forests model. Taxa in red belong to the phylum Proteobacteria and taxa in green are butyrate producing Firmicutes.

Similar to the tissue-specific insulin sensitivity, we ordered each dataset in quartiles of fasting glucose and insulin, HOMA-IR, HOMA-B and HbA_1c_ and used random forest classifiers to determine whether the bacterial composition was related to these markers, by using the highest and lowest quartiles as classes (supplementary Table 2). Random Forests is a supervised machine learning technique, which can utilize nonlinear relationships and complex dependencies between genus-like groups to identify bacterial taxa that differentiate the faecal community composition of individuals that are in the highest or lowest range of each host parameter, respectively. The measure of the success of the method is its ability to classify samples correctly. Random classification (always choosing 1 class would yield a classification error of 0.5, therefore, the performance should be higher if the input values or predictors (relative abundance of bacterial taxa) assist in classification. Only MAA showed a moderate improvement over random classification for individuals with the highest and lowest 25% of fasting glucose concentrations with an Area Under the Curve (AUC) of 0.66 or HbA_1c_ 0.65. with taxa from *Clostridium* clusters IV (*Faecalibacterium prausnitzii* et rel. and *Butyrivibrio crossatus* et rel.) and XIVa (*Roseburia intestinalis, Clostridium nexile and Eubacterium rectale* and related species) and several Proteobacteria *(Enterobacter aerogenes* et rel. and *Vibrio*) as the most important microbiota prediction features for classification of individuals with high or low fasting glucose concentrations (Fig. 4). For HbA_1c_ similar bacterial orders were among the most important taxa. However, different members of these higher phylogenetic groups were identified, such as for *Clostridium* clusters IV (*Oscillospira guillermondii* et rel., *Sporobacter termitidis* et rel., *Faecalibacterium prausnitzii* et rel.) and IX (*Peptococcus niger* et rel.) as well as Proteobacteria (*Novosphingobium*, *Vibrio* and *Aeromonas*) (Fig. 4).

## Discussion

The aim of this cross-sectional study was to determine the relation between microbiota composition and tissue-specific insulin-sensitivity in two independent cohorts of obese males with ranging levels of insulin resistance. To our knowledge, this is the first observational study that considers hyperinsulinemic-euglycemic clamp-derived data, known to be the gold standard for determination of insulin sensitivity. In two independent cohorts of obese and overweight subjects, we assessed peripheral, hepatic and adipose tissue insulin sensitivity using a two-step hyperinsulinemic-euglycemic clamp in combination with a [6,6-^2^H_2_]-glucose tracer infusion. Only after correcting for several covariates, we found some associations between microbial taxa and adipose tissue, liver or skeletal muscle insulin sensitivity. However, the pattern of correlations was very distinct for each cohort and even showed significant associations with an opposite direction in each cohort. This might indicate that predicting the role of microbiota based on baseline compositional data in host insulin sensitivity is challenging when the obese state has already developed. In line with this, the significant correlations between surrogate measures of insulin sensitivity and glycemic control and gut microbiota composition, were very different in both cohorts, with even opposite directions of correlations. This showed that we could not identify a similar pattern of correlations between MAA and AMS or conserved associations between bacterial abundance and markers of tissue-specific insulin sensitivity (e.g. consistent identical direction of a correlation between a taxon with a metabolic parameter in both cohorts), because both cohorts showed highly divergent patterns. For instance, several *Bacteroides* species (*vulgatus*, *intestinales* and *ovatus*) showed conflicting correlations with hepatic insulin sensitivity (% suppression of EGP) in both cohorts, highlighting the challenges associated with baseline observations in a single (non-intervention) cohort.

To gain more insight into these differing patterns we applied random forest classification of quartiled host parameters. Overall, in both cohorts the microbiota composition at genus level showed performance that was close to random classification, with differences in predictors as well as classification accuracy between these cohorts. To this end, only MAA showed a moderate improvement over random classification for fasting glucose and HbA_1c_. The most important microbiological feature for the prediction of HbA_1c_ in MAA was the abundance of *Peptococcus niger* et rel. whose association was also identified using linear regression, underscoring the need for complementary analytical approaches. Among important microbiota features were several taxa that are known to produce butyrate^18^. These observations are in line with previous studies that showed a decreased abundance of some universal butyrate-producing bacteria and an increase in *Lactobacillus* and various opportunistic pathogens, associated with type 2 diabetes in a cohort of European women^5,6^ and Chinese individuals^9^. More importantly these authors also showed that the discriminant metagenomic markers for type 2 diabetes differed between the cohorts and concluded that, metagenomic predictive tools for type 2 diabetes should be specific for the age and geographical location of the populations studied^5^. The obvious differences in observations between the cohorts and the fact that the identified associations of peripheral and hepatic insulin sensitivity and other markers of glucose homeostasis, with specific gut microbiota members were very distinct for each cohort, illustrate that findings may differ from one cohort to another and confirm the observations by Karlsson and co-authors^5^.

Remarkably, before adjusting for any covariates in both cohorts no relationships between microbial composition and tissue-specific insulin sensitivity, as determined by state-of-the-art clamp techniques, were detected in both cohorts. Only after correction for covariates, such as age, BMI and waist/hip ratio numerous significant associations (q < 0.2) were found. Previous studies have indicated a differential microbial composition in type 2 diabetes or impaired glucose tolerant subjects, but this was based on comparisons with individuals that exhibited normal glucose tolerance^5,6,9,19^. In contrast, the individuals in this study were all overweight, showed impaired glucose metabolism (impaired fasting glucose and/or impaired glucose tolerance) or had the metabolic syndrome, therefore it might be possible that the role of the microbiota is altered when a metabolically compromised state has already developed, thus playing a more prominent role in the initial development of an impaired metabolic health than in the further development towards type 2 diabetes and cardiometabolic complications. Nevertheless, our populations exhibited a wide range of (tissue-specific) insulin sensitivity, ranging from normal to a considerably impaired insulin sensitivity.

On top of these observations, there were striking differences between the microbiota compositions of both cohorts, which were mainly linked to a subset of individuals (Fig. 2). Compared to AMS, the abundance of bifidobacteria was higher MAA, which has been linked to a healthier phenotype, a reduction in inflammatory markers and an improvement in glucose homeostasis and lipid metabolism^20,21^. However, a random forests based predictive model for type 2 diabetes, identified several *Bifidobacterium* species as highly predictive and enriched in type 2 diabetes in a Chinese, but not in a Swedish cohort^5^. Another difference between both cohorts was a lower abundance of specific taxa belonging to *Clostridium* cluster XIVa in MAA, which included known butyrate producers (e.g. *Butyrivibrio crossotus* and *Clostridium symbiosum* and related species)^18^. Also, there were several slight differences in metabolic profiles between both cohorts, with lower triacylglycerol (TAG) and glucose concentrations as well as a reduced HOMA-B in MAA. Although lower abundances of butyrate-producing bacteria have previously been reported in type 2 diabetes subjects in various states of insulin resistance^5^, these compositional and metabolic differences were not linked in a significant way.

The strikingly cohort-specific observations suggest that the relation between microbiota composition and type 2 diabetes as well as other characteristics of the metabolic syndrome is very dependent on the selected cohort of patients and their respective baseline microbiota composition. Similar observations have been made by other researchers as well^5,11^. In addition, it could be that alterations in microbiota composition are differently associated with the insulin resistance phenotype when the overweight and/or obese state of the patient is already established, as is the case for our metabolic syndrome patients. In the latter case we cannot exclude that the composition of the fecal microbiota may play a role in the worsening of insulin sensitivity in an early stage in the development from a lean towards an overweight/obese phenotype.

With regards to the difference in composition between the cohorts, the human microbiota is highly individual, which we also clearly observed in our PCA plot (Fig. 2). Although we know its composition is impacted by numerous external as well as host-specific factors, including diet, age, antibiotics use, BMI, gender, and genotype^22,23^, the interplay of variables that give rise to the variation of the microbiota is not yet fully understood because of its complexity and the influence of numerous stochastic variables, such as common exposures over a timeframe of years^22^. Nevertheless, the multitude of these variations and their combinations may explain why individual-specific (dysbiotic) microbiota profiles are continuously observed.

This individuality of the microbiota composition may also explain why fecal transplantation from lean donors to recipients with the metabolic syndrome slightly improved peripheral insulin sensitivity only in a subgroup of subjects (metabolic responders), whereas other individuals did not show any effect on these parameters upon the intervention^15^. A follow-up study consisting of the individuals of the AMS cohort presented here, confirmed these observations in a larger population, however the effect was also shown to be transient, as both the microbiota and the metabolic parameters returned to baseline 18 weeks post transplant. Furthermore, the authors identified a panel of bacterial species whose abundance at baseline could predict the responder status with high accuracy^16^. Although the driving factors are currently unknown, they speculate that the level of metabolic response might be due to specific donor-host interactions. This shows that a difference of associations of microbial taxa with metabolic features between cohorts, does not automatically mean that a treatment for subsets of individuals, such as microbial transplant with a specific composition that are needed to shift an individual microbial ecosystem towards eliciting a metabolic response, cannot be predicted based on baseline composition.

The strength of the present study is the detailed phenotyping of study participants regarding glucose homeostasis and insulin sensitivity, since we evaluated tissue specific insulin sensitivity with hyperinsulinemic-euglycemic clamp-derived data. Limitation of this study are its cross-sectional design limiting causal implications and a lack of information on other microbiota covariates such as diet.

Our study demonstrated that longitudinal studies are required to investigate the relation between fecal microbiota composition and insulin sensitivity in phenotypes varying in adiposity and insulin sensitivity. Such studies should also address microbial function to reveal if a microbial- or host component, or both can be found with predictive power.

Currently, we have no clear explanation of the remarkable difference between the two cohorts with respect to microbiota composition as well as the associations between microbial profiles and host metabolic parameters. Both cohorts included subjects with an increased risk for type 2 diabetes mellitus and/or metabolic syndrome in geographically closely located areas in the Netherlands (~150 km distance). It is striking that the general microbiota compositional variation of MAA only partly overlapped with AMS and that a subgroup-specific microbiota was only observed in MAA. We hypothesize that a subset of the metabolic syndrome patients of this cohort exhibit an alternative state of microbiota composition that is driven by a yet unknown force which is only present in this cohort. Nevertheless, this study clearly demonstrates that cohort-specific microbiota differences hamper finding a consensus biological interpretation between studies based on single baseline cohort observations. This, combined with the complexity of individual disease pathogenesis, as well as the individual-specific differences in microbiota composition, may explain the inconsistency in observations between different studies concerning the identification of signature microbes for obesity, as well as other disorders and diseases including inflammatory bowel disease and irritable bowel syndrome^24–26^.

## Conclusion

In the present study, tissue-specific insulin sensitivity, at the level of the adipose tissue, liver and skeletal muscle showed cohort specific correlations with the abundance of microbial genus-level groups in two cohorts of obese, insulin resistant males at baseline. With respect to the surrogate measures for insulin sensitivity and measures of glycemic control HbA_1c_ and fasting glucose, the fecal microbiota composition showed predictive potential, but only in one of the cohorts. These latter findings stress the importance of taking metabolic profiles, environmental, genetic and microbial variables into account in future studies. Overall, our data combining detailed information on microbial composition and the insulin sensitivity phenotype in two cohorts, indicated that predictions regarding the role for gut microbiota in host tissue-specific insulin sensitivity after development of the obese, insulin resistant state are difficult to extrapolate based on baseline microbiota composition alone.

## Methods

### Study population

We investigated baseline microbiota composition in relation to tissue-specific insulin sensitivity and other indicators of glucose metabolism in two independent cohorts of overweight and obese (BMI 25–45 kg/m^2^) Caucasian men between 35–70 years old (ClinicalTrials.gov NCT02241421 and Dutch Trial Register NTR2705). The cohort from Maastricht (MAA) consisted of 56 low-active (<3 hr organized sports activities per week), weight-stable (<2 kg body weight change 3 months prior to inclusion) subjects with impaired fasting glucose levels (IFG, glucose concentration ≥ 5.6 mmol/l) and/or impaired glucose tolerance (IGT, 2 h plasma glucose during a 75 g oral glucose tolerance test 7.8–11.1 mmol/l. Exclusion criteria were the use of antibiotics for a period of 3 months before entering the study; cancer; liver malfunction, known allergic reactions to any type of antibiotics; hearing disorders; major illnesses with a life expectancy less than 5 years and pulmonary hepatic, cardiovascular, kidney, and gastrointestinal disease. Subjects did not use lipid- and glucose-lowering drugs, beta-blockers, anti-oxidants or chronic corticosteroids^17^. The cohort from Amsterdam (AMS) consisted of 42 subjects diagnosed with the metabolic syndrome according to the NCEP criteria: (≥3/5: fasting plasma glucose ≥ 5.6 mmol/l, triglycerides ≥ 1.7 mmol/l, waist-circumference> 102 cm, high-density lipoprotein (HDL-) cholesterol <1.03 mmol/l, blood pressure ≥ 130/85 mmHg), were treatment naive and otherwise healthy^16,27^. Exclusion criteria were a cardiovascular event, history of recent weight loss, cholecystectomy and the use of any medication known to influence gut microbial composition in the last three months (including antibiotics and pre-/pro-/synbiotics and proton pump inhibitors) or targeting metabolic diseases (e.g., anti-diabetic, lipid-lowering and/or drugs). Subjects were recruited by newspaper advertisement in their consecutive regions and gave written informed consent before participation after reading the study protocol. For the study from Amsterdam the protocol was reviewed and approved by the Institutional Review Board of the Academic Medical Center (AMC) in Amsterdam, the Netherlands and conducted at the AMC. The study was registered at the Dutch Trial Register (number 2705). For the study from Maastricht the protocol was reviewed and approved by the local Medical Ethical Committee of Maastricht University Medical Center+ and registered under ClinicalTrials.gov NCT02241421. All procedures were performed according to the declaration of Helsinki (revised version, 2008, Seoul, South Korea).

### Study Design

Study measurements were conducted following a 10 h overnight fast. The primary outcome of this study was tissue-specific insulin sensitivity (insulin-mediated glucose disposal (Rd)), hepatic insulin sensitivity (insulin-mediated suppression of endogenous glucose production (% suppression EGP)), adipose tissue insulin sensitivity (insulin-mediated suppression of plasma free fatty acids (% suppression FFA)) as determined by a two-step hyperinsulinemic-euglycemic clamp with [6,6-^2^H_2_]-glucose infusion. To this end, one cannula was inserted into the antecubital vein, whereas a second Teflon cannula was inserted into a superficial dorsal hand vein for blood sampling, which was arterialized by placing the hand into a hot-box, blowing warm air (~50 °C). In the Maastricht cohort, after a bolus-injection of 2.4 mg kg^−1^ was infused, continuous tracer-infusion was started at 0.04 mg kg^−1^ min^−1^ and continued throughout the measurement. After 2 h, low-dose insulin was infused at 10 mU m^−2^ min^−1^ for 2 h^28^, followed by high-dose insulin at 40 mU m^−2^ min^−1^ for 2 h. By variable co-infusion of a 17.5%-glucose solution, enriched by 1.1% [6,6−^2^H_2_]-glucose-tracer^29^, plasma concentrations were maintained at 5.0 mmol/l. In the cohort  from Amsterdam, insulin was infused at 20 mU m^-2^ min^−1^ for 2 h, followed by 60 mU m^−2^ min^−1^ for 2 h^30^. For calculation of steady-state-kinetics, the last 30 minutes of each step (0, 10 and 40mU m^−2^ min^−1^ insulin) and the last 20 minutes of each step (0, 20 and 60 mU m^−2^ min^−1^ insulin), respectively, were used for the two cohorts, during which additional blood samples were taken.

In addition, we collected fasting plasma samples to determine insulin and glucose concentrations for the calculation of the homeostasis model assessment for insulin resistance (HOMA-IR, [(fasting insulin (μIU/ml) × fasting glucose (mmol/l))/22.5)] and β-cell function (HOMA-B, [20 × (fasting insulin)/(fasting glucose – 3.5)]. Anthropometrical measurements were performed for the calculation of the body mass index (BMI, [weight (kg)/height(m)^2^] and waist/hip ratio.

### Biochemical analysis

Blood was collected into pre-chilled tubes, centrifuged at 1000 *g*, and plasma was snap-frozen and stored at −80 °C until analyses. Isotopic enrichment of plasma glucose was determined by electron ionization gas chromatography–mass spectrometry and expressed as tracer-to-tracer ratio for steady-state calculations of rate of disappearance (Rd) and endogenous glucose production (EGP). Plasma glucose and glycerol were determined with the Cobas Fara auto-analyzer, Roche, Switzerland). Plasma insulin was measured with a double antibody radioimmunoassay (Millipore, MA, USA), and plasma FFA concentrations were analyzed using standard enzymatic techniques automated on a Cobas Fara auto-analyzer (Roche).

### Fecal microbiota characterization

The fecal microbiota composition was determined by analyzing 16S rRNA gene amplicons using the Human Intestinal Tract Chip Microarray (HITChip); a phylogenetic microarray targeting the V1 and V6 hypervariable regions of the 16S rRNA gene of over 1000 intestinal bacterial phylotypes^31^. DNA was isolated from faeces using the repeated bead beating method as previously described,^32^ and subsequently used for microbiota profiling. In short, full length 16S rRNA genes were amplified by PCR using primers T7prom-Bact-27-for and Uni-1492-rev^31^, followed by *in vitro* transcription, Cy3/Cy5 labelling and fragmentation of RNA, and hybridization. The signal intensity data from the microarray hybridizations were collected from the Agilent G2505C scanner (Agilent Technologies) using the Agilent Feature Extraction software, version 10.7.3.1 and pre-processed using an in-house MySQL database and custom R scripts. Each scanner channel from the array was spatially normalized separately using polynomial regression, followed by outlier detection and filtering in each set of probes with a χ2 test. Duplicate hybridizations with a Pearson correlation >98% were considered for further analysis, and microbiota profiles were generated by pre-processing of probe-level measurements with min-max normalization and RPA probe summarization^33^ into three phylogenetic levels: order-like, genus-like (>90% sequence similarity), and phylotype-like (>98% sequence similarity)^31^. In the present study the analysis focused on the genus-level variation, referred to as species and relatives (‘et rel.’). Log10-transformed signals were used as a proxy for bacterial abundance.

### Statistical analysis

To determine the bacterial groups whose relative abundance significantly differed between the two cohorts, a non-paired Wilcoxon test was used. We performed Spearman correlations and partial Spearman correlations (adjusted for age, BMI and waist/hip ratio) using the *ppcor* package^36^ to associate specific genus-like bacterial groups with the variables under investigation in both cohorts separately. All outcome variables were sorted into quartiles. The highest and lowest range were used for logistic regression and Random Forest classification using the Random Forest and ROCR R packages^34,35^. For all analyses Benjamini–Hochberg (BH) correction was applied for multiple testing (q-value) and values of q < 0.2 were considered significant. Missing data were removed before any analysis. All analyses were performed in R, v3.4.0^37^. Principal component analysis was performed in Canoco v5^38^ with log10 transformed signals summarized to genus like groups.

### Accession codes

The phylogenetic HITChip microarray data matrix and anonymized subject metadata are available: 10.6084/m9.figshare.11341985

### Disclosure

The research is funded by TI Food and Nutrition (project GH003), a public-private partnership on pre-competitive research in food and nutrition. The funders had no role in study design, data collection and analysis, decision to publish, or preparation of the manuscript.

## Supplementary information

Dataset 1.
