# Supplementary material for: Individual and cohort-specific gut microbiota patterns associated with tissue-specific insulin sensitivity in overweight and obese males
Source: Sci Rep. 2020 May 5;10:7523. doi: 10.1038/s41598-020-64574-4 (PMC7200728; doi:10.1038/s41598-020-64574-4)
Supplement: Supplementary file 1 — Dataset 1. [file 41598_2020_64574_MOESM1_ESM.docx]

***Supplementary Data***

***Individual and cohort-specific gut microbiota patterns associated with tissue-specific insulin sensitivity in overweight and obese males.***

Gerben D.A. Hermes^1,2,*^ , Dorien Reijnders^2,3,*^ , Ruud S. Kootte^2,4^, Gijs H. Goossens^2,3^, Hauke Smidt^1,2^, Max Nieuwdorp^2,4^, Ellen E. Blaak^2,3,$^, Erwin G. Zoetendal^1,2,$^

**Supplementary Table 1.** A complete overview of the differential abundance of all detected genus like groups.

| **L1 (Phylum/Class)** | **L2 (Genus like)** | **Mean MAA (SD)** | **Mean AMS (SD)** | **Corrected**  **pval** |
| --- | --- | --- | --- | --- |
| Actinobacteria | Bifidobacterium | 4.09 (0.59) | 3.58 (0.39) | 0.001 |
| Clostridium cluster IV | Sporobacter termitidis et rel. | 3.79 (0.45) | 4.18 (0.52) | 0.004 |
| Actinobacteria | Propionibacterium | 2.45 (0.51) | 2.11 (0.1) | 0.001 |
| Clostridium cluster III | Clostridium stercorarium et rel. | 2.83 (0.36) | 3.16 (0.36) | 0.003 |
| Uncultured Clostridiales | Uncultured Clostridiales II | 3.43 (0.36) | 3.76 (0.42) | 0.003 |
| Bacteroidetes | Bacteroides vulgatus et rel. | 3.87 (0.5) | 3.57 (0.51) | 0.018 |
| Clostridium cluster IV | Subdoligranulum variable at rel. | 4.44 (0.42) | 4.73 (0.48) | 0.008 |
| Clostridium cluster XIVa | Clostridium nexile et rel. | 3.77 (0.42) | 4.05 (0.28) | 0.009 |
| Clostridium cluster XIVa | Butyrivibrio crossotus et rel. | 3.75 (0.33) | 4.02 (0.24) | 0.001 |
| Clostridium cluster IV | Papillibacter cinnamivorans et rel. | 3.54 (0.37) | 3.8 (0.27) | 0.004 |
| Proteobacteria | Burkholderia | 1.99 (0.28) | 1.73 (0.1) | 2.46E-08 |
| Actinobacteria | Collinsella | 3.35 (0.7) | 3.1 (0.37) | 0.274 |
| Clostridium cluster IV | Clostridium cellulosi et rel. | 4.15 (0.53) | 4.38 (0.51) | 0.118 |
| Clostridium cluster XIVa | Bryantella formatexigens et rel. | 3.81 (0.39) | 4.04 (0.24) | 0.02 |
| Clostridium cluster XIVa | Eubacterium ventriosum et rel. | 3.59 (0.61) | 3.81 (0.36) | 0.146 |
| Clostridium cluster IV | Anaerotruncus colihominis et rel. | 2.97 (0.33) | 3.19 (0.37) | 0.006 |
| Clostridium cluster XIVa | Lachnobacillus bovis et rel. | 3.53 (0.38) | 3.75 (0.23) | 0.016 |
| Clostridium cluster XIVa | Dorea formicigenerans et rel. | 4.23 (0.36) | 4.45 (0.2) | 0.016 |
| Clostridium cluster XIVa | Clostridium symbiosum et rel. | 4.06 (0.37) | 4.27 (0.24) | 0.011 |
| Uncultured Clostridiales | Uncultured Clostridiales I | 3.42 (0.39) | 3.62 (0.44) | 0.047 |
| Clostridium cluster IV | Clostridium orbiscindens et rel. | 4 (0.39) | 4.2 (0.27) | 0.043 |
| Clostridium cluster XVII | Catenibacterium mitsuokai et rel. | 2.02 (0.3) | 2.22 (0.38) | 1.00E-03 |
| Bacteroidetes | Bacteroides uniformis et rel. | 2.72 (0.51) | 2.53 (0.5) | 0.118 |
| Clostridium cluster XIVa | Roseburia intestinalis et rel. | 3.36 (0.47) | 3.55 (0.26) | 0.105 |
| Proteobacteria | Alcaligenes faecalis et rel. | 2.42 (0.3) | 2.23 (0.11) | 0.004 |
| Clostridium cluster IX | Peptococcus niger et rel. | 2.3 (0.15) | 2.48 (0.31) | 0.001 |
| Clostridium cluster XIVa | Eubacterium rectale et rel. | 3.76 (0.41) | 3.93 (0.24) | 0.118 |
| Bacteroidetes | Parabacteroides distasonis et rel. | 3.18 (0.36) | 3 (0.37) | 0.036 |
| Clostridium cluster IX | Dialister | 2.71 (0.49) | 2.88 (0.53) | 0.084 |
| Actinobacteria | Eggerthella lenta et rel. | 2.83 (0.39) | 2.65 (0.16) | 0.118 |
| Clostridium cluster XIVa | Ruminococcus gnavus et rel. | 3.64 (0.46) | 3.81 (0.29) | 0.218 |
| Clostridium cluster IV | Clostridium leptum et rel. | 3.74 (0.48) | 3.9 (0.42) | 0.246 |
| Proteobacteria | Sutterella wadsworthia et rel. | 2.92 (0.26) | 2.76 (0.17) | 0.009 |
| Clostridium cluster XIVa | Ruminococcus lactaris et rel. | 3.29 (0.58) | 3.43 (0.47) | 0.247 |
| Clostridium cluster XIVa | Anaerostipes caccae et rel. | 3.91 (0.45) | 4.05 (0.36) | 0.264 |
| Clostridium cluster IV | Oscillospira guillermondii et rel. | 4.16 (0.58) | 4.3 (0.59) | 0.492 |
| Clostridium cluster IV | Ruminococcus callidus et rel. | 3.73 (0.46) | 3.87 (0.37) | 0.246 |
| Proteobacteria | Oxalobacter formigenes et rel. | 2.97 (0.42) | 2.84 (0.31) | 0.281 |
| Clostridium cluster XIVa | Outgrouping clostridium cluster XIVa | 3.84 (0.46) | 3.97 (0.4) | 0.292 |
| Clostridium cluster IX | Mitsuokella multiacida et rel. | 2.2 (0.44) | 2.32 (0.43) | 0.016 |
| Clostridium cluster XIVa | Lachnospira pectinoschiza et rel. | 4.16 (0.44) | 4.27 (0.29) | 0.534 |
| Bacilli | Aneurinibacillus | 1.71 (0.07) | 1.8 (0.12) | 1.00E-03 |
| Clostridium cluster I | Clostridium (sensu stricto) | 3.2 (0.29) | 3.3 (0.25) | 0.036 |
| Bacteroidetes | Bacteroides intestinalis et rel. | 2.04 (0.3) | 1.94 (0.3) | 0.214 |
| Clostridium cluster IX | Megasphaera elsdenii et rel. | 2.48 (0.26) | 2.57 (0.36) | 0.247 |
| Proteobacteria | Enterobacter aerogenes et rel. | 2.84 (0.19) | 2.75 (0.11) | 0.016 |
| Clostridium cluster XIVa | Coprococcus eutactus et rel. | 4.71 (0.39) | 4.63 (0.3) | 0.246 |
| Proteobacteria | Xanthomonadaceae | 2.06 (0.11) | 1.98 (0.1) | 0.001 |
| Clostridium cluster XV | Anaerofustis | 1.84 (0.11) | 1.92 (0.11) | 0.02 |
| Bacteroidetes | Bacteroides ovatus et rel. | 2.93 (0.27) | 2.86 (0.25) | 0.281 |
| Bacteroidetes | Prevotella oralis et rel. | 3.1 (0.86) | 3.17 (0.73) | 0.493 |
| Bacilli | Enterococcus | 2.58 (0.41) | 2.5 (0.09) | 0.776 |
| Clostridium cluster IV | Eubacterium siraeum et rel. | 2.36 (0.21) | 2.43 (0.21) | 0.039 |
| Bacteroidetes | Prevotella melaninogenica et rel. | 3.71 (0.98) | 3.79 (0.85) | 0.603 |
| Actinobacteria | Actinomycetaceae | 2.01 (0.2) | 1.94 (0.1) | 0.374 |
| Bacilli | Weissella et rel. | 2.18 (0.18) | 2.25 (0.18) | 0.022 |
| Clostridium cluster XIVa | Clostridium sphenoides et rel. | 4 (0.35) | 4.06 (0.23) | 0.879 |
| Clostridium cluster XV | Eubacterium limosum et rel. | 2.12 (0.09) | 2.19 (0.12) | 0.004 |
| Verrucomicrobia | Akkermansia | 2.12 (0.33) | 2.06 (0.26) | 0.776 |
| Bacteroidetes | Tannerella et rel. | 2.85 (0.2) | 2.79 (0.26) | 0.118 |
| Bacilli | Streptococcus intermedius et rel. | 2.5 (0.28) | 2.55 (0.26) | 0.235 |
| Bacilli | Lactobacillus salivarius et rel. | 2.13 (0.21) | 2.19 (0.29) | 0.055 |
| Clostridium cluster XVI | Bulleidia moorei et rel. | 2.24 (0.05) | 2.29 (0.1) | 0.009 |
| Clostridium cluster XVIII | Coprobacillus catenaformis et rel. | 2.5 (0.19) | 2.55 (0.2) | 0.326 |
| Bacteroidetes | Prevotella tannerae et rel. | 2.6 (0.22) | 2.55 (0.31) | 0.142 |
| Uncultured Mollicutes | Uncultured Mollicutes | 2.82 (0.18) | 2.87 (0.18) | 0.08 |
| Bacilli | Bacillus | 2.07 (0.05) | 2.12 (0.09) | 0.003 |
| Bacilli | Streptococcus mitis et rel. | 3.32 (0.57) | 3.36 (0.48) | 0.667 |
| Proteobacteria | Campylobacter | 2.47 (0.03) | 2.51 (0.09) | 0.003 |
| Fusobacteria | Fusobacteria | 2.54 (0.04) | 2.59 (0.09) | 0.006 |
| Clostridium cluster XVII | Lactobacillus catenaformis et rel. | 1.88 (0.07) | 1.92 (0.11) | 0.006 |
| Spirochaetes | Brachyspira | 1.86 (0.03) | 1.9 (0.1) | 0.017 |
| Proteobacteria | Oceanospirillum | 2.19 (0.05) | 2.23 (0.12) | 0.264 |
| Clostridium cluster XI | Clostridium difficile et rel. | 3.59 (0.55) | 3.55 (0.47) | 0.899 |
| Proteobacteria | Haemophilus | 1.87 (0.03) | 1.9 (0.09) | 0.028 |
| Clostridium cluster XIII | Peptostreptococcus micros et rel. | 2.28 (0.03) | 2.32 (0.09) | 0.043 |
| Bacteroidetes | Bacteroides fragilis et rel. | 3.01 (0.3) | 2.97 (0.37) | 0.493 |
| Clostridium cluster XVI | Eubacterium cylindroides et rel. | 2.29 (0.05) | 2.33 (0.1) | 0.027 |
| Proteobacteria | Proteus et rel. | 2.49 (0.05) | 2.53 (0.09) | 0.018 |
| Proteobacteria | Bilophila et rel. | 2.1 (0.06) | 2.13 (0.11) | 0.131 |
| Proteobacteria | Helicobacter | 2.27 (0.03) | 2.31 (0.09) | 0.047 |
| Clostridium cluster XIVa | Clostridium colinum et rel. | 2.79 (0.36) | 2.82 (0.31) | 0.246 |
| Bacteroidetes | Bacteroides plebeius et rel. | 2.85 (0.29) | 2.81 (0.34) | 0.507 |
| Proteobacteria | Klebisiella pneumoniae et rel. | 2.52 (0.16) | 2.48 (0.1) | 0.527 |
| Bacilli | Lactobacillus plantarum et rel. | 3.02 (0.25) | 2.99 (0.22) | 0.711 |
| Clostridium cluster XIVa | Ruminococcus obeum et rel. | 4.89 (0.33) | 4.92 (0.3) | 0.885 |
| Clostridium cluster XVI | Eubacterium biforme et rel. | 2.59 (0.43) | 2.62 (0.29) | 0.286 |
| Proteobacteria | Desulfovibrio et rel. | 2.32 (0.06) | 2.35 (0.1) | 0.246 |
| Proteobacteria | Moraxellaceae | 1.88 (0.04) | 1.91 (0.1) | 0.264 |
| Clostridium cluster IX | Phascolarctobacterium faecium et rel. | 2.63 (0.3) | 2.66 (0.27) | 0.243 |
| Bacilli | Streptococcus bovis et rel. | 3.58 (0.59) | 3.61 (0.5) | 0.589 |
| Proteobacteria | Yersinia et rel. | 2.22 (0.05) | 2.25 (0.1) | 0.281 |
| Clostridium cluster IV | Faecalibacterium prausnitzii et rel. | 4.88 (0.45) | 4.91 (0.51) | 0.762 |
| Bacilli | Lactococcus | 2.15 (0.23) | 2.17 (0.15) | 0.264 |
| Bacteroidetes | Allistipes et rel. | 3.3 (0.36) | 3.28 (0.41) | 0.776 |
| Clostridium cluster XVIII | Clostridium ramosum et rel. | 2.41 (0.18) | 2.43 (0.13) | 0.342 |
| Clostridium cluster IV | Ruminococcus bromii et rel. | 3.78 (0.7) | 3.79 (0.59) | 0.96 |
| Clostridium cluster XI | Anaerovorax odorimutans et rel. | 3.29 (0.33) | 3.27 (0.24) | 0.947 |
| Clostridium cluster IX | Megamonas hypermegale et rel. | 2.12 (0.23) | 2.14 (0.24) | 0.145 |
| Actinobacteria | Corynebacterium | 2.08 (0.04) | 2.1 (0.09) | 0.77 |
| Bacilli | Granulicatella | 1.8 (0.3) | 1.81 (0.17) | 0.105 |
| Bacilli | Staphylococcus | 1.97 (0.13) | 1.95 (0.09) | 0.973 |
| Bacteroidetes | Prevotella ruminicola et rel. | 2.02 (0.13) | 2.03 (0.15) | 0.933 |
| Clostridium cluster XIVa | Eubacterium hallii et rel. | 3.97 (0.41) | 3.98 (0.38) | 0.986 |
| Proteobacteria | Pseudomonas | 1.91 (0.12) | 1.92 (0.1) | 0.264 |
| Bacilli | Wissella et rel. | 1.96 (0.25) | 1.95 (0.25) | 0.858 |
| Proteobacteria | Vibrio | 2.3 (0.1) | 2.31 (0.1) | 0.589 |
| Bacteroidetes | Bacteroides splachnicus et rel. | 2.95 (0.12) | 2.94 (0.2) | 0.264 |
| Clostridium cluster IX | Veillonella | 2.32 (0.16) | 2.31 (0.15) | 0.933 |
| Bacilli | Lactobacillus gasseri et rel. | 2.82 (0.29) | 2.82 (0.22) | 0.118 |
| Bacteroidetes | Uncultured Bacteroidetes | 1.9 (0.28) | 1.9 (0.26) | 0.885 |
| Proteobacteria | Escherichia coli et rel. | 2.8 (0.17) | 2.8 (0.2) | 0.561 |
| Actinobacteria | Atopobium | 1.96 (0.09) | 1.96 (0.11) | 0.733 |
| Bacteroidetes | Bacteroides stercoris et rel. | 2.6 (0.18) | 2.6 (0.21) | 0.872 |

**Supplementary Table 2.** Quartile boundaries for markers of insulin sensitivity

| **MAA** |  |  |  |  |
| --- | --- | --- | --- | --- |
|  | Q1 | Q2 | Q3 | Q4 |
| HOMA-IR | [2.24,3.14] | (3.14,3.98] | (3.98,5.55] | (5.55,8.72] |
| HOMA-B | [63.8,94.8] | (94.8,122] | (122,153] | (153,286] |
| fasting insulin | [8.8,11.8] | (11.8,15.5] | (15.5,19.7] | (19.7,29.3] |
| fasting glucose | [4.7,6.1] | (6.1,7.1] | (7.1,8.6] | (8.6,11.2] |
| HbA_1c_ | [29,36.1] | (36.1,37.7] | (37.7,40.4] | (40.4,49.7] |
| %EGP | [17.6,31.1] | (31.1,41.9] | (41.9,47.9] | (47.9,75.7] |
| Rd | [10.7,15.7] | (15.7,24.9] | (24.9,28.3] | (28.3,51.4] |
| %FFA | [-6.1,33.6] | (33.6,44] | (44,59.8] | (59.8,84.1] |
|  |  |  |  |  |
| **AMS** |  |  |  |  |
|  | Q1 | Q2 | Q3 | Q4 |
| HOMA-IR | [1.68,3.6] | (3.6,5.13] | (5.13,6.22] | (6.22,10.8] |
| HOMA-B | [55.8,128] | (128,163] | (163,270] | (270,468] |
| fasting insulin | [6.5,15.3] | (15.3,19.7] | (19.7,25.2] | (25.2,42.3] |
| fasting glucose | [4.38,5.37] | (5.37,5.62] | (5.62,6.08] | (6.08,7] |
| HbA_1c_ | [30,36.5] | (36.5,39] | (39,42] | (42,46] |
| %EGP | [30.8,46.3] | (46.3,54.9] | (54.9,61.8] | (61.8,85] |
| Rd | [10.1,19.1] | (19.1,25.1] | (25.1,33.1] | (33.1,43.9] |
| %FFA | [53.9,68.8] | (68.8,73.9] | (73.9,81] | (81,92.1] |

**Supplementary Figure 1.** Within cohort microbiota similarity of MAA and AMS calculated by Pearson correlations.
